# Genomics in Egypt: Current Status and Future Aspects

**DOI:** 10.3389/fgene.2022.797465

**Published:** 2022-05-13

**Authors:** Eman Ahmed El-Attar, Rasha Mohamed Helmy Elkaffas, Sarah Ahmed Aglan, Iman S. Naga, Amira Nabil, Hoda Y. Abdallah

**Affiliations:** ^1^ Chemical Pathology Department, Medical Research Institute, Alexandria University, Alexandria, Egypt; ^2^ Clinical and Chemical Pathology Department, Faculty of Medicine, Cairo University, Cairo, Egypt; ^3^ Department of Microbiology, Medical Research Institute, Alexandria University, Alexandria, Egypt; ^4^ Department of Human Genetics, Medical Research Institute, Alexandria University, Alexandria, Egypt; ^5^ Medical Genetics Unit, Histology and Cell Biology Department, Faculty of Medicine, Suez Canal University, Ismailia, Egypt; ^6^ Center of Excellence in Molecular and Cellular Medicine, Faculty of Medicine, Suez Canal University, Ismailia, Egypt

**Keywords:** genomics, Egypt, cancer, communicable disease, COVID 19, governance

## Abstract

Egypt is the third most densely inhabited African country. Due to the economic burden and healthcare costs of overpopulation, genomic and genetic testing is a huge challenge. However, in the era of precision medicine, Egypt is taking a shift in approach from “one-size-fits all” to more personalized healthcare via advancing the practice of medical genetics and genomics across the country. This shift necessitates concrete knowledge of the Egyptian genome and related diseases to direct effective preventive, diagnostic and counseling services of prevalent genetic diseases in Egypt. Understanding disease molecular mechanisms will enhance the capacity for personalized interventions. From this perspective, we highlight research efforts and available services for rare genetic diseases, communicable diseases including the coronavirus 2019 disease (COVID19), and cancer. The current state of genetic services in Egypt including availability and access to genetic services is described. Drivers for applying genomics in Egypt are illustrated with a SWOT analysis of the current genetic/genomic services. Barriers to genetic service development in Egypt, whether economic, geographic, cultural or educational are discussed as well. The sensitive topic of communicating genomic results and its ethical considerations is also tackled. To understand disease pathogenesis, much can be gained through the advancement and integration of genomic technologies via clinical applications and research efforts in Egypt. Three main pillars of multidisciplinary collaboration for advancing genomics in Egypt are envisaged: resources, infrastructure and training. Finally, we highlight the recent national plan to establish a genome center that will aim to prepare a map of the Egyptian human genome to discover and accurately determine the genetic characteristics of various diseases. The Reference Genome Project for Egyptians and Ancient Egyptians will initialize a new genomics era in Egypt. We propose a multidisciplinary governance system in Egypt to support genomic medicine research efforts and integrate into the healthcare system whilst ensuring ethical conduct of data.

## Introduction

Egypt is a densely occupied country with a total population exceeding 100,388,000, a life expectancy of 71.8 years and a healthy life expectancy of 63 years. Maternal mortality ratio approaches 37 per 100 000 live births. Approximately 26.2% of the population has health expenditures of more than 10% of household income. Both the basic health sector and medical research receive around 0.10 US dollars per capita as official development assistance. Under-five mortality rate is 20 per 1000 live births. These figures delineate the huge economic burden of healthcare costs due to overpopulation. In spite of all challenges, universal health coverage (service coverage index) reached 66 out of 100 in 2017 (World Health Organization, 2021).

Ever since the first draft of the Human genome project was published in 2001 and fully completed and published in 2003, the genomic field has been enormously growing, with huge discoveries and great applications improving healthcare and managing diseases that were once before deemed incurable. Genetic diseases are of particular concern in Egypt owing to the high consanguinity rate in the population (Shawky et al., 2011). Congenital genetic defects were noticed as anomalies thousands of years ago, since the discovery of sculptures and mummies in temples revealed some inherited disorders as those observed in Tutankhamun; cleft palate, oligodactyly, clubfoot (Hawass et al., 2010) as well as evidence of dwarfism, osteogenesis imperfecta and others that could be seen in mummies (Kozma, 2008).

This article presents the status of genomic knowledge, services, applications, needs, regulation and population acceptance in Egypt. It highlights some important challenges and efforts to overcome them, shedding the light on more aspects that need to be addressed and fulfilled.

### Diseases and Health Indices in Egypt

Disease burden is the main driver for healthcare service prioritization and research efforts. In Egypt, several diseases are of particular concern such as hepatitis, specifically hepatitis C viral (HCV) infection, which was estimated to be 4.5—6.7% prevalent in 2016 (World Health Organization, 2016). A huge national screening program was initiated in 2018 for screening of the population for HCV and optimal treatment requires a great effort to eliminate the disease from Egypt (Abdel-Razek et al., 2019). Chronic respiratory, cardiovascular diseases, diabetes mellitus and cancer account for 28% of the mortality causes in 2019. It was reported that cancer's five-year prevalence was 278165, with new cases reaching 134632 in 2020. Liver cancer, the most reported among cancer cases, accounted for 20.7% of all cancer types and it is the most reported in males as well. Breast cancer comes next (16.4%) as the first among cancers that affect females, followed by non-Hodgkin lymphoma (5.4%), bladder cancer (7.9%) and lung cancer (4.9%) (Iarc.fr, 2022).

### Current Need for Genomic Medicine and Applications in Egypt

#### Burden of Genetic Diseases

Genetic diseases have been reported to affect 2–5% of all live births. Two main factors lead to increased genetic disease burden in Egypt including old maternal age and consanguineous marriages.

Although the rate of consanguineous marriage is decreasing worldwide, most Middle Eastern Arabs, still have the custom of preferring consanguineous marriage (Hamamy and Alwan, 1994). Shawky et al., 2011 revealed that the overall frequency of consanguinity within Egyptians is around 35% which is considered quite high; however, this frequency varies by region (59.9% in rural areas & 17% in urban areas) (Shawky et al., 2011). The adverse health effects of consanguineous marriage include a greater risk not only on producing offspring which are homozygous for a deleterious recessive gene variant, but also individuals with increased susceptibility for polygenic disorders, still births, spontaneous abortions, child, or neonatal deaths, as well as congenital anomalies (Capmas, 2022). There is also a noticeable relationship between consanguinity and specific physical defects, behavior and psychiatric disorders (Bittles, 2001; Shawky et al., 2011). Accordingly, consanguinity represents a risk factor and a public health concern in Egypt for many unfavorable outcomes (Shawky et al., 2011). Variable genetic disorders are frequent in Egypt. Neurological disorders are the most diagnosed genetic disorders among Egyptian children followed by neuromuscular diseases, skeletal anomalies and inborn errors of metabolism as reported by Shawky et al., 2012 (Shawky et al., 2012) and by Afifi et al., 2010 (Afifi et al., 2010).

#### Availability and Accessibility to Genetic Services and Research Centers

The awareness of the importance of medical genetics in Egypt was well appreciated in the early 1960s by establishing the first medical genetics units at the pediatric departments of Cairo and Ain Shams Universities (https://www.asu.edu.eg/ce/84/page) (Temtamy and Hussen, 2017) In 1966, the specialty of Human genetics at the National Research Centre (NRC) (https://www.nrc.sci.eg/human-genetics-genome-research-division/) was established and in 1967, the medical genetics unit at the Medical Research Institute (MRI) in Alexandria commenced (https://mri.alexu.edu.eg/). This was followed by the establishment of medical genetics units in many other universities (Temtamy et al., 2010). Those centers include multiple specialized teams covering variable genetic disorders and offer genetic counseling, clinical diagnosis of patients with genetic disorders, follow up for patients with known or suspected genetic disorders and other supportive services for patients and their families, with a registry for genetic disorders including thousands of cases. To date, more than 21,000 families with rare genetic disorders or seeking genetic counseling, are registered since 1981 in the Medical Research Institute in Alexandria alone. It is noteworthy that there are continuous international collaborations between the aforementioned Institutions; NRC and MRI and research teams worldwide in attempt to reach the correct diagnosis and unravel the etiology and pathogenesis of rare disorders and identify causative mutations implicated in rare genetic syndromes. These research studies are either conducted on a cohort or per individual case and many of the past studies solved many cases and led to very promising and interesting results in the studied rare genetic syndromes in different genetic subspecialities; neuromuscular disorders, growth disorders, ciliopathies, ocular anomalies, congenital heart diseases, skeletal disorders, dental genetic abnormalities, inherited metabolic disorders, craniofacial disorders, dermatological anomalies and other variable rare genetic disorders with multiple congenital anomalies or disorders with underlying chromosomal aberrations or microdeletions (Toomes et al., 1999; Zaki et al., 2007; Bielas et al., 2009; Handley et al., 2013; Rice et al., 2013; Traverso et al., 2013; Abdalla et al., 2014; Novarino et al., 2014; Abdalla et al., 2015; El-Hattab et al., 2016; Scott et al., 2016; Seifi et al., 2016; Abdalla et al., 2017; Fassad et al., 2018; Maddirevula et al., 2018; Patel et al., 2018; Fassad et al., 2020a; Fassad et al., 2020b; Chatron et al., 2020; Nabil et al., 2020; Shamseldin et al., 2020; Donato et al., 2021; Essawi et al., 2021; Horn et al., 2021; Meyer et al., 2021; Patel et al., 2021; Thomas et al., 2021). Moreover, several clinical genetic studies on categories of syndromes or rare disorders were conducted in Egypt. One example is the study conducted by Shawky et al., 2010 on a cohort of patients with limb anomalies to reveal the prevalence of isolated limb anomalies versus those with well-defined genetic syndromes or chromosomal aberrations (Shawky et al., 2022). Also comprehensive studies of the clinical, cytogenetic or molecular aspects of some rare disorders as Robinow syndrome and Roberts syndrome were acknowledged (Aglan et al., 2015; Ismail et al., 2016).

In 2002, the first national committee for community genetic services was settled and its main mission was setting prevention and management policy guidelines of genetic disorders. In the same year, the committee established a national genetic counseling program. The main objective was promoting free of charge genetic services. The program involved a hierarchical system of referrals from primary health care (PHC) to secondary care, then tertiary care level of services. The Ministry of Health and Population (MoH&P) started delivering primary and secondary care services through health facilities, where early detection is done at PHC level, followed by referral of cases to the secondary care services which include genetic counseling clinics in the catchment area to be consulted by well-trained secondary care physicians who offer diagnosis, counseling, treatment and referral for rehabilitation services. Complex cases are then referred to tertiary care provided through university and institutional genetic departments and units (Raouf, 2008).

Public services are also provided by non-governmental organizations (NGOs) for those who have genetic disorders at a reasonable cost. Moreover, private laboratories provide testing services involving several technologies including and not limited to cytogenetic, molecular genetic tests, metabolic diagnostic tests for screening inborn errors of metabolism. Some of the tests are either performed in those private laboratories or outsourced for the lack of technology or high cost and low demand, rendering the test highly expensive. In addition to the aforementioned centers offering genetic services, other centers offer research services as well. The Molecular biology Research and Diagnostics unit (MRDU) in the Clinical and Chemical Pathology Department, Cairo University (https://clinchemd.kasralainy.edu.eg/clinchem-labs is one of the top-notch research facilities for postgraduate studies. The Genomics Centre (GC) in Zewail city (https://zewailcity.edu.eg/) employs a multidisciplinary approach to better understand how genomes function in health and disease. Aswan Heart Centre (https://myf-egypt.org/aswan-heart-centre/) is one of the largest heart centers in Egypt and the Middle East. The center aims at defining the Egyptian genetic landscape, linking genotypic, phenotypic data based on individual levels for cardiovascular disorders (Shawky et al., 2012).

The Center of Excellence in Molecular & Cellular Medicine (http://fom.suez.edu.eg/) at Suez Canal University, Ismailia, serves genomic research starting from diagnosis to treatment in the area of Eastern Egypt including the Suez Canal area and Sinai. Although there is increased awareness for the need of genetic services, a gap between the need and the availability is present. In 2008, it was reported that only a small percentage (20%) of more than 4000 primary health facilities across the country offer the services of detection and referral of genetic disorders and only 11 genetic counseling clinics were distributed over 7 out of 27 governorates (Cairo, Giza, Alexandria, Portsaid, Elsharquia, ElMenia, Assiut) (Raouf, 2008). Unfortunately, there are no updated data on the current situation. A growingly dense population as in Egypt mandates continuous efforts to extend and expand service delivery as well as updating diagnostic technology to meet healthcare needs. Thorough planning for accessibility, extension of testing services and availability of advanced technologies at a reasonable cost and/or full coverage by insurance is of utmost importance. Thus, the role of national genomic medicine initiatives is to address these barriers and challenges (Stark et al., 2019).

#### Newborn Screening

Early detection of diseases is crucial for their management. In 1991, the first newborn screening for hypothyroidism in Egypt was conducted by human genetics experts from Cairo university, Medical Research Institute of Alexandria, Ain Shams and Mansoura universities. In 1996, the first newborn screening for metabolic disorders in Alexandria governorate took place through a study conducted by the Medical Research Institute where newborns were screened for three treatable inborn errors of metabolism, namely phenylketonuria “PKU”, galactosemia and congenital hypothyroidism, aiming at early detection of these diseases and providing therapeutic regimens to guard against development of mental retardation (Ismail et al., 1996). Collectively, all the conducted studies have created an important awareness among physicians, scientists, decision makers, and the public about the importance of human genetics as a medical science in Egypt. The results of this neonatal screening of 15,000 newborns showed a high frequency of Phenylketonuria (1:7000), and of hypothyroidism (1:3000) (Temtamy, 1998), which convinced the health authorities in Egypt to start a mass neonatal screening program for hypothyroidism in all Egyptian governorates in 2003; screening for PKU was later added as well (Temtamy, 2019).

Rare diseases face the challenges of being appropriately diagnosed, receiving adequate care and affording personalized medications (Stoller, 2018). However, a landmark in raising awareness on rare diseases among the public was in August 2021, since a presidential initiative to treat muscular atrophy with the government bearing its high cost and urged the early diagnosis of the disease. This implied the establishment of a national registry for spinal muscular atrophy and encouraged many genetic laboratories in the public and private sector to offer genetic testing for different types of the diseases (State Information Service, 2022).

#### Non-Communicable Diseases (NCDs) Genomics Initiatives

In 2018, NCDs were reported to be responsible for 84% of deaths of the Egyptian population with cardiovascular diseases (CVDs) representing the most common cause of mortality (40%) and cancer was next (13%). NCD-related premature mortality is strikingly high and affecting young people in initiating their economic independence through associated disabilities which frequently ends with death (World Health Organization, 2022). According to a review performed as part of the development of a framework for implementation of public health genomics in Africa, it is estimated that these dramatic figures are mostly due to changes in lifestyle associated with urbanization and sedentary lifestyle together with intake of high-salt and high-lipid diets as well as genetic factors (Policy paper: A framework for the implementation of genomic medicine for public health in Africa, 2022). A collaborative work between Egypt Ministry of health and population and the World Health Organisation (WHO) had resulted in developing a five-years multisectoral action plan for NCD prevention and control (2018–2022), with a primary goal to achieve a 15% reduction in premature mortality from NCDs by 2022 (Egyptian Ministry of Health and Population and World Health Organization, 2022).

### Cardiac Genomics

CVDs are heterogeneous pathological conditions which have complex genetic etiology as well as environmental-molecular interactions. In ancient Egypt, atherosclerotic vascular disease had been reported after thorough examination of many mummies in various studies, one of them studied 44 mummies and showed that nearly half had evidence atherosclerosis (Butrous et al., 2020).

The role of genetics in CVDs in populations of African ancestry has been recently studied in a number of projects (Scott et al., 2016; Owolabi et al., 2019) However, the need for a national reference population data to identify the healthy state and to use it in variant interpretation was addressed in 2016 by the Egyptian Collaborative Cardiac Genomics (ECCO-GEN) Project through the Aswan Heart Center and the Magdi Yacoub Global Heart Foundation support. The project aimed to recruit 1,000 Egyptian volunteers free of CVDs (EHVols) from the local population and to identify genetic variation in genes previously shown to be involved in inherited cardiac conditions (ICCs), especially those involved in hypertrophic cardiomyopathy (HCM) and dilated cardiomyopathy (DCM) (Aguib et al., 2020). The first 391 samples of an Egyptian healthy volunteers cohort from the EHVol study were sequenced using a targeted panel and 1262 variants were identified in 27 cardiomyopathy genes, their sequencing data were deposited on the European Genome-Phenom Archive (EGA) under the accession code: EGAD00001006160 and the dataset was published on May 20,2020. The ECCO-GEN project aims at providing individual-level genetic and phenotypic data to support future studies in CVD and population genetics.

In 2020, a pilot study was published that investigated sarcomeric and non-sarcomeric variants in a cohort of idiopathic pediatric hypertrophic cardiomyopathy patients using next generation sequencing and suggested that due to the population’s high consanguinity burden, a particular genetic background contributes significantly to pediatric hypertrophic cardiomyopathy patients in Egypt (Darwish et al., 2020).

### Cancer

According to the WHO Globocan 2020 cancer report about cancer prevalence in Egypt, the commonest sites for cancers were the liver (20.7%) and breast (16.4%) for both sexes (Cancer today, 2022). Ibrahim et al. developed a mathematical model and estimated a 3-fold increase in cancer incidence in Egypt by 2050 compared to 2013 (Ibrahim et al., 2014). These figures have drawn the attention of the Egyptian authorities to prioritizing cancer as a national health problem that needs more efforts among both research and services sectors. The genetic background of cancer has directed Egyptian researchers concerned with this discipline to focus more on genetics and genomics. The Egyptian population is a heterogeneous population (Tishkoff et al., 2009) and accordingly this necessitates implementing genomic/genetic studies to reflect the diversity among its different subpopulations. In this section, we will shed the light on genetic/genomic research efforts relating to most prevalent two types of cancers in Egypt: breast and liver cancers.

### Breast Cancer and the Egyptian Women's Health Initiative

**FIGURE 1:**
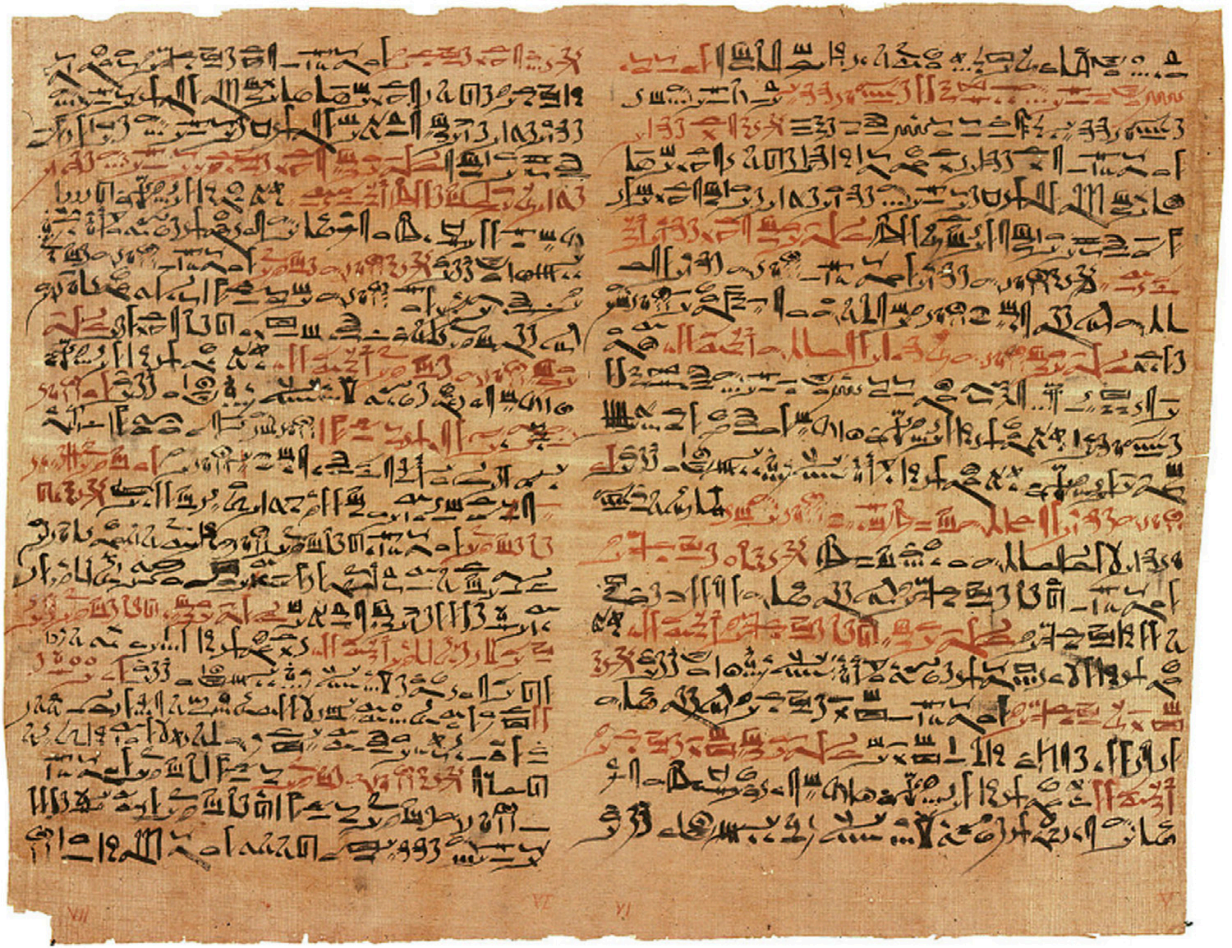
Edwin Smith papyrus (Image credit — U.S. National Library of Medicine).

Breast cancer (BC) history in Egypt dates to around 3000 BC in the ancient Egyptian medical documents - the Edwin Smith Papyrus, the first and oldest cancer record as shown in Figure 1 (Ades et al., 2017). It stated that bulging lesions of the breast were a grave disease, and the ancient Egyptians tried to treat these lesions with cautery, knives, and salts.

The earliest genomic research on BC in Egypt focused on unraveling the role of genetic variations in the tumor suppressor genes *BRCA1/2* and *TP53* (El- A Helal et al., 2000; Hussein and Hassan, 2004; Swellam et al., 2004; Saleh et al., 2004) Other research focused on identification of novel *BRCA1*/2 variants and their role in early-onset and sporadic breast and/or ovarian cancer in Egypt (Abdel-Mohsen et al., 2016). As the studies on the *BRCA1/2* genes have expanded, new population-based large genomic rearrangements have been revealed (Hagag et al., 2013; Eid et al., 2017). Considering *TP53* variant research, several researchers initially focused on a common codon 72 (Hussein and Hassan, 2004; Saleh et al., 2004; El-Ghannam et al., 2011). For other DNA repair genes, *XPD* was the most studied one in Egypt (Hussien et al., 2012; Ramadan et al., 2014). Although *BRCA1*/2 are the most studied genes in Egyptian cancer research, clearly there is still a knowledge gap in determining the mutational effects that determine disease incidence and clinical outcomes in BC. Epigenetic aspects have been studied as important contributors to the risk and prognosis of BC reported in Egypt, including the role of microRNAs in BC tumor tissues, circulating free miRNA, circulating long non-coding RNA (Hafez et al., 2012; Zidan et al., 2018) in addition to DNA methylation status of known susceptibility genes such as *FHIT* (Zaki et al., 2015).

As a part of the country’s endeavors for advancing Egyptian population health, the President’s “Egyptian Women's Health Initiative” was launched in 2020 to promote the health of Egyptian women (Presidency.eg, 2022). This initiative was a major step against the alarming increase in BC among Egyptian women aiming at screening 28 million women in all Egyptian governorates; emphasizing on regular self-examination; and providing free-of-charge treatment.

However, due to the high expenses of these testing procedures, BC genetic testing could not be provided in this initiative. Genetic testing services for BC are mostly available via the private sector or non-governmental organizations (NGOs), and is utilized in BC prevention, recurrence risk prediction, metastatic BC and BC survivors. Examples of bodies and private labs providing this service are the Breast Cancer Foundation of Egypt, Genetic Diagnostic Center, Hassan Healthcare LLC and Generations Genetic Laboratory.

### Liver Cancer

Liver cancer (LC) is the most prevalent cancer among males in Egypt (Ibrahim et al., 2014). It is reported that chronic inflammation of the liver like non-alcoholic fatty liver disease (NAFLD), non-alcoholic steatohepatitis (NASH), and liver cirrhosis are major predisposing factors for the development of hepatocellular carcinoma (HCC) in Egypt. Viral hepatitis is an important causative agent of these liver diseases finally leading to LC in Egypt (Rotimi et al., 2020).

Most of the genetic studies on LC in Egypt focused on the contribution of *TP53* expression on different genetic mutations, and other tumor suppressor genes like *TP73, RB, KLF6, and CTNNB1*, to liver carcinogenesis (El-Kafrawy et al., 2005; El Far et al., 2006; Wahab et al., 2010).

Regarding epigenetics studies, Egyptian researchers have focused on discovering novel biomarkers for LC like microRNAs. These studied the role of serum miRNA-224, miRNA-215, miRNA-143, miRNA-122, miRNA-199a, and miRNA-16 (El-Abd et al., 2015; Mamdouh et al., 2017). Motawi et al. reported the contributing role of miRNA-122 and miRNA-222 as a discriminating biomarker for distinguishing liver injury from LC (Motawi et al., 2016). They also unraveled the possible role of the genetic variants LncRNA HULC rs7763881 and MALAT rs619586 in the progression of hepatitis virus-persistent carriers’ progression to LC (Motawi et al., 2019).

### Communicable Diseases and SARS-CoV-2 Research Efforts

#### Coronavirus disease 2019

The Corona virus disease 2019 “COVID-19” started in Wuhan, China by the end of December 2019 (Shigemura et al., 2020) and has quickly spread throughout the world (Ojha et al., 2020). The causative agent of this infectious disease of the respiratory tract is Severe Acute Respiratory Syndrome Coronavirus 2 “SARS-CoV-2” (Lake, 2020). On March 11^th^, 2020, COVID-19 was declared a pandemic by the WHO.

The importance of disease monitoring systems and genome sequencing technology in improving public health has been highlighted by COVID-19 (Institute of Pathogen Genomics, 2020). In Africa a total of 140 disease outbreaks are anticipated to occur each year (World Health Organization, 2020) which are additional to the threats of endemic infectious diseases, which make up approximately 35 percent of Africa's 10 million annual fatalities (Roser and Ritchie, 2016). These figures highlight the crucial need for Africa's epidemic preparedness and surveillance systems to be expanded, including genomic and digital surveillance capabilities (Eib.org, 2020) and manufacturing capabilities for diagnostics and treatments in the local area (Nkengasong, 2020).

Pathogen genomics can revolutionize public health surveillance through enhancing outbreak detection, following up on transmission routes, searching for genetic changes that affect infectivity, diagnosis, treatments, and vaccinations, as well as evaluating the efficacy of interventions (Armstrong et al., 2019). As a consequence, public health experts will be able to stay on top of new micro-organisms and re-emerging diseases. It will enable health systems to more effectively combat pathogens that cause diseases such as COVID-19, polio, malaria, tuberculosis, HIV/AIDS, cholera, Ebola, and other emerging health issues as well as antibiotic resistance (Institute of Pathogen Genomics, 2020).

Rapid advances in sequencing technology have led to development of dependable next-generation sequencing (NGS) equipment that can resolve pathogens at a high resolution. Despite the considerable need to reduce Africa's substantial burden of infectious diseases, NGS application is limited (Inzaule et al., 2021). The capacity for sequencing in Africa is scant and limited. About 71% of next-generation sequencers are clustered in just five African countries: South Africa (*n* = 79; 38%), Kenya (*n* = 28; 14%), Nigeria (*n* = 13; 6%), Morocco (*n* = 18; 9%), and Egypt (*n* = 10; 5%). Sanger-based assays are used in the majority of African countries. Due to the high prices of reliable NGS equipment and techniques, financial constraints, very few resources and inadequate infrastructure and framework for data-sharing, its full potential has yet to be realized in Africa. Democratizing the use of NGS technologies in Africa and other low- and middle-income regions will enable partnerships and overturn the habit of relying on partners from the global north for cutting-edge global health innovation (Institute of Pathogen Genomics, 2020).

Africa CDC Institute of Pathogen Genomics, through the Africa Pathogen Genomics Initiative (Africa PGI) intends to improve disease surveillance and public health collaborations (Institute of Pathogen Genomics, 2020). Various approaches are being implemented in collaboration with the Africa CDC-led African Task Force for Coronavirus Preparedness and Response (AFTCOR) laboratory technical working group.

First, resources are being mobilized to speed SARS-CoV-2 sequencing across Africa, exploiting the continent's available next-generation sequencing capabilities, and 16 countries are receiving resources and technical assistance. Second, a network of sequencing laboratories, genomics, and bioinformatics professionals is in the making to pool resources, information and expertise. Third, policies are being created to ensure that data is representative and repeatable. Fourth, sample referral methods are being developed with regional laboratories to network countries having insufficient sequencing capacity (Tessema et al., 2020).

SARS-CoV-2 has evolved over time, and genomic data has been used to identify and track nosocomial infection spread in South Africa (Giandhari et al., 2021), sources of introduction and lineages (Tessema et al., 2020), including the emergence and disperse of more transmissible variants with the potential to affect disease activity and countermeasures such as vaccines (Toyoshima et al., 2020; World Health Organization, 2016).

Omicron variant of SARS-CoV-2 has been first reported in Egypt December the 18^th^ 2021 in three cases (Al-Youm, 2021). In order to respond more quickly to the Omicron variant and the increase in cases, WHO is assisting countries in improving genomic surveillance to trace the virus and find additional potential variants that could arise. A regional genome sequencing laboratory in South Africa is assisting 14 southern African countries and has considerably increased sequencing capacity. Southern African countries sequenced only 5500 samples in the first half of 2021. They're currently sequencing the same number every month (World Health Organization, 2022).

The first country in the Middle East to be struck by the COVID-19 pandemic was Iran, followed by Turkey, Saudi Arabia, Qatar, and Egypt (Temtamy et al., 2010). Although the number of cases was low in the beginning, it has accelerated at an unprecedented rate, hitting all Middle Eastern countries. This could be a reflection of the readiness and response measures taking place in Middle Eastern countries. The Middle East, which sits at the crossroads of Eurasia and Africa, has played a significant role in shaping modern civilizations and religions. It is a highly sensitive area in terms of geography, economics, politics, culture, and religion (Baloch et al., 2020). There are significant obstacles in limiting the spread of COVID-19 in the Middle East. Compromised healthcare systems, long-term regional conflicts and humanitarian catastrophes, lack of transparency and cooperation, and frequent religious gatherings are just a few examples. These variables are intertwined, and their combined effect determines how this region responds to the epidemic (Baloch et al., 2020).

In Egypt, from January 3^rd^, 2020 to September 3^rd^, 2021, there have been 288,732 cases of COVID-19 with 16,743 deaths, reported to WHO and as of August 27^th^, 2021, a total of 8,741,005 vaccine doses have been given (WHO (COVID-19) Dashboard with Vaccination Data, 2022).

Using next generation sequencing; interpretation of viral genome sequences presents insight about the pattern of distribution around the world, the genetic diversity during epidemics and pandemics as well as the kinetics of the development of different subtypes. SARS-CoV-2 database, such as ”GISAID (www.gisaid.org)” and the “NCBI SARS-CoV-2 database (https://www.ncbi.nlm.nih.gov/sars-cov-2)” allow the genomic data, as well as epidemiological data for the sequenced isolates, to be made public (Zekri et al., 2021a).

Until September 8^th^,2021 on GISAID index.php, there are 977 genomes shared on GISAID from Egypt. Few genomic studies were conducted in Egypt due to the high economic burden.

**FIGURE 2:**
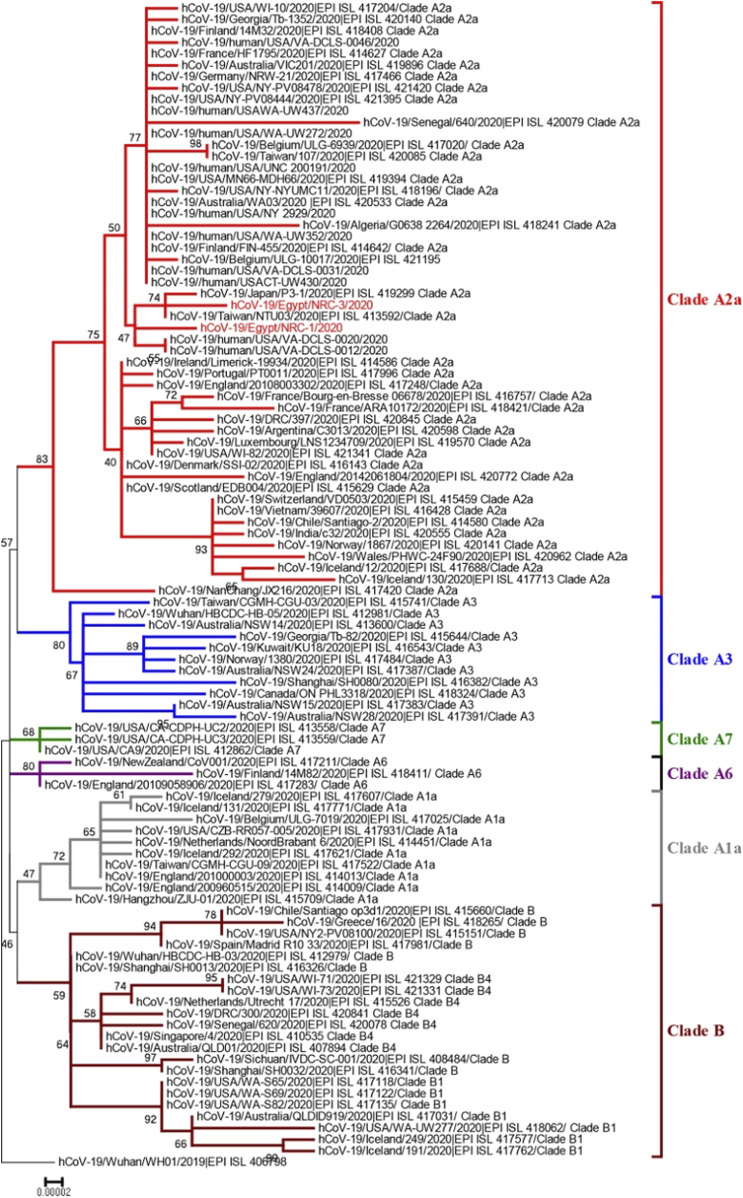
Neighbor-joining phylogenetic tree of a SARS-CoV-2 strain from Egypt and other global strains. The two Egyptian strains are shown in red. The percentage of replicate trees in which the associated taxa clustered together in the bootstrap test (1,000 replicates) is shown at the dendrogram nodes. The phylogenetic analysis was performed using MEGA7 (Kandeil et al., 2020). Source: Coding-Complete Genome Sequences of Two SARS-CoV-2 Isolates from Egypt. Permission Request ID: 600053988.

In a study by Kandeil et al. (Kandeil et al., 2020) in May 2020 they announced the genome sequences of two SARS-CoV-2 isolates found in patients from Egypt “The isolate hCoV-19/Egypt/NRC-1/2020 and hCoV-19/Egypt/NRC-3/2020”. These sequences are available at the GISAID-EpiCoV newly emerging coronavirus SARS-CoV-2 platform with the listed identifiers “EPI_ISL_430819 and EPI_ISL_430820” available at https://www.gisaid.org/. These strains belong to clade A2a, which cover strains from different countries like Asia, Europe, the United States, Australia, and Africa (Figure 2). Another study by Zekri et al. (Zekri et al., 2021a) in November 2020 conducted a study on 61 whole genome sequences from Egyptian patients. They detected 204 unique sequence variations in Egyptian patients’ genomes. It was concluded that most Egyptian genomic strains sequenced were close to isolates originating from the United States of America, Austria, Sweden, Saudi Arabia and France.

Further work by Zekri et al. (Zekri et al., 2021b) in April 2021 used next-generation sequencing to search for mutation hotspots in Egyptian COVID-19 patients and identify dominant variations that may be linked to differences in clinical presentations. They concluded that D614G/spike-glycoprotein and P4715L/RNA-dependent-RNA polymerase were the most common mutations among Egyptian isolates. These mutations were linked to transmissibility regardless of symptom changeability. E3909G-nsp7 could answer why children recover so quickly. Nsp6-L3606fs, spike-glycoprotein-V6fs, and nsp13-S5398L variants may be associated with clinical manifestations intensifying.

Furuse Y et al. (Furuse, 2021) in his recent article stated that forty-nine countries have already published >100 SARS-CoV-2 genomic sequences with large number of all published genomic sequences (*n* = 93,817) originating from United Kingdom (38.9%) and United States (22.7%). However, only two sequences originated from Egypt which emphasizes the need to increase sequencing capacities and funding possibilities.

Egypt's death rate from infectious diseases has gradually decreased, from 20.87 percent in 2000 to 9.63 percent in 2019 (Knoema.com, 2022). Some of the major communicable diseases in Egypt include Typhoid fever, hepatitis virus and tuberculosis.

There are huge efforts dedicated to genomic technology in the field of microbiology in Egypt. The microbiology field has been completely altered using genomics. Genomic analyses are yielding unparalleled insights into microbial evolution and diversity and are explaining the complexity of the genetic variation in both host and pathogens that underlies disease (Knoema.com, 2019).

In Egypt, Shoeib et al. (Shoeib et al., 2020) performed whole-genome analysis on a fecal specimen obtained from a hospitalized 6-month-old child with acute gastroenteritis in 2012. They unexpected rotavirus group A (RVA) strain “RVA/Human-tc/EGY/AS997/2012/G9 [14]”. Phylogenetic analysis designated that the strain AS997 had the consensus P[14] genotype cluster with G9, T1 and H1 reassortment. This finding proposes either a mixed gene arrangement developed from a human Wa-like strain with a P[14]-containing animal virus, or that this P[14] was obtained only through the reassortment of human strains. The study highlights the significance of whole-genome analysis in surveillance studies of rotavirus strains and reveals the potential roles of interspecies transmission and numerous reassortments resulting in the development of new rotavirus genotypes.

#### Hepatitis viruses

Viral hepatitis is a major public health issue and a cause of mortality in Egypt. Around 8–10 millions are living with viral hepatitis in Egypt (Centers for Disease Control and Prevention, 2012; Globalhep.org, 2014). The most common causes of viral hepatitis in Egypt are Hepatitis A virus (HAV) and hepatitis E virus (HEV). By the age of 15, approximately half of the Egyptian population had been infected with HAV. Moreover, more than 60% of the Egyptian population are anti‐HEV positive in the first ten years of life. Hepatitis B virus (HBV), hepatitis C virus (HCV), and hepatitis D virus (HDV) are the most significant causes for chronic hepatitis, liver cirrhosis, and hepatocellular carcinoma (HCC) in Egypt (Elbahrawy et al., 2021).

Egypt has a moderate endemic level for HBV with only around 3.3 million people infected. In 1992 after the initiation of mass immunization program for infants in Egypt; the prevalence rate has declined (Sherif et al., 1985). Statistically significant decrease in anti‐HCV prevalence has been reported among the general population ranging from 0 to 51% (median value: 13%) (Kouyoumjian et al., 2018) The prevalence of HCV was 8.7–40.3% (Kandeel et al., 2017; Bayomy Helal et al., 2018; El-Ghitany and Farghaly, 2019; Soliman et al., 2019) before the National HCV treatment program was carried out. A large‐scale national study conducted during 2018–2019 outlined a much lower rate of HCV prevalence (4.6%) (Waked et al., 2020).

In 2016, 194 member states of the WHO had devoted themselves by 2030 viral hepatitis will no longer be a public health hazard, focusing mainly on HBV and HCV infection. A “National Committee for the Control of Viral Hepatitis (NCCVH)” was established by the MOH&P in 2006. By April 2008, this committee had developed a national control strategy for viral hepatitis, which mandated improvements in preventive actions regarding reducing the incidence of HBV and HCV infections and offered more efficient treatment accessibility for patients with chronic hepatitis. Egypt held the largest mass screening and treatment campaign for HCV in late 2018, which allowed screening 50 million people for HCV infection as a step toward disease eradication (Waked et al., 2020).

#### Tuberculosis

Tuberculosis (TB) is another major public health threat worldwide, competing with HIV as the cause of death due to infectious diseases worldwide (Sulis et al., 2014). According to the WHO, around 8.6 million TB cases were estimated in 2012. Most cases are in Asia and Africa (58 and 27%, respectively) (World Health Organization, 2013). TB is still a health problem in Egypt, affecting the young active age group. TB is a significant challenge to public health officials, particularly “drug-resistant (DR) and multidrug-resistant (MDR)” isolates of *Mycobacterium tuberculosis*. Alyamani et al. (Alyamani et al., 2019) reported that around 35% of TB isolates are MDR in Egypt which is relatively high. Phylogenetic and molecular dating analyses showed that lineages coming from Egypt recently diverged (∼78 years). In contrast to drug-sensitive isolates, drug-resistant isolates are not clustered or largely disseminating, proposing deficient treatment as the main reason for antimicrobial resistance emergence rather than higher virulence or more capacity to survive.

We are witnessing nowadays a revolution in sequencing technology rendering it rapid, and robust. The advancement in this technology impacted greatly COVID-19 global response. The rapid sequence data sharing has allowed countries to develop assays for detection, helped in deeper understanding of pathogenesis and better prediction of action as well as fast production of vaccines. It has become clear in this pandemic that global pathogen genomic surveillance strategy is needed indeed to enhance preparedness for pathogens with pandemic or epidemic potentials. The WHO has taken an initiative setting a draft for Global genomic surveillance strategy and invited various stakeholders; academia, member states, national health authorities, industry, and civil societies to review the draft in December 2021. The draft represents an important step towards empowering public health actions in response to pandemics and epidemics around the world, supporting and encouraging collaborative work and data and specimens sharing (World Health Organization, 2022). Genomic surveillance for pathogens is still costly for many countries including Egypt. However, given its impact on public and global health, governments should advocate pathogen genomic surveillance establishment through direction of funds towards capacity building and training.

### Challenges and Barriers to Genomic Testing

**FIGURE 3:**
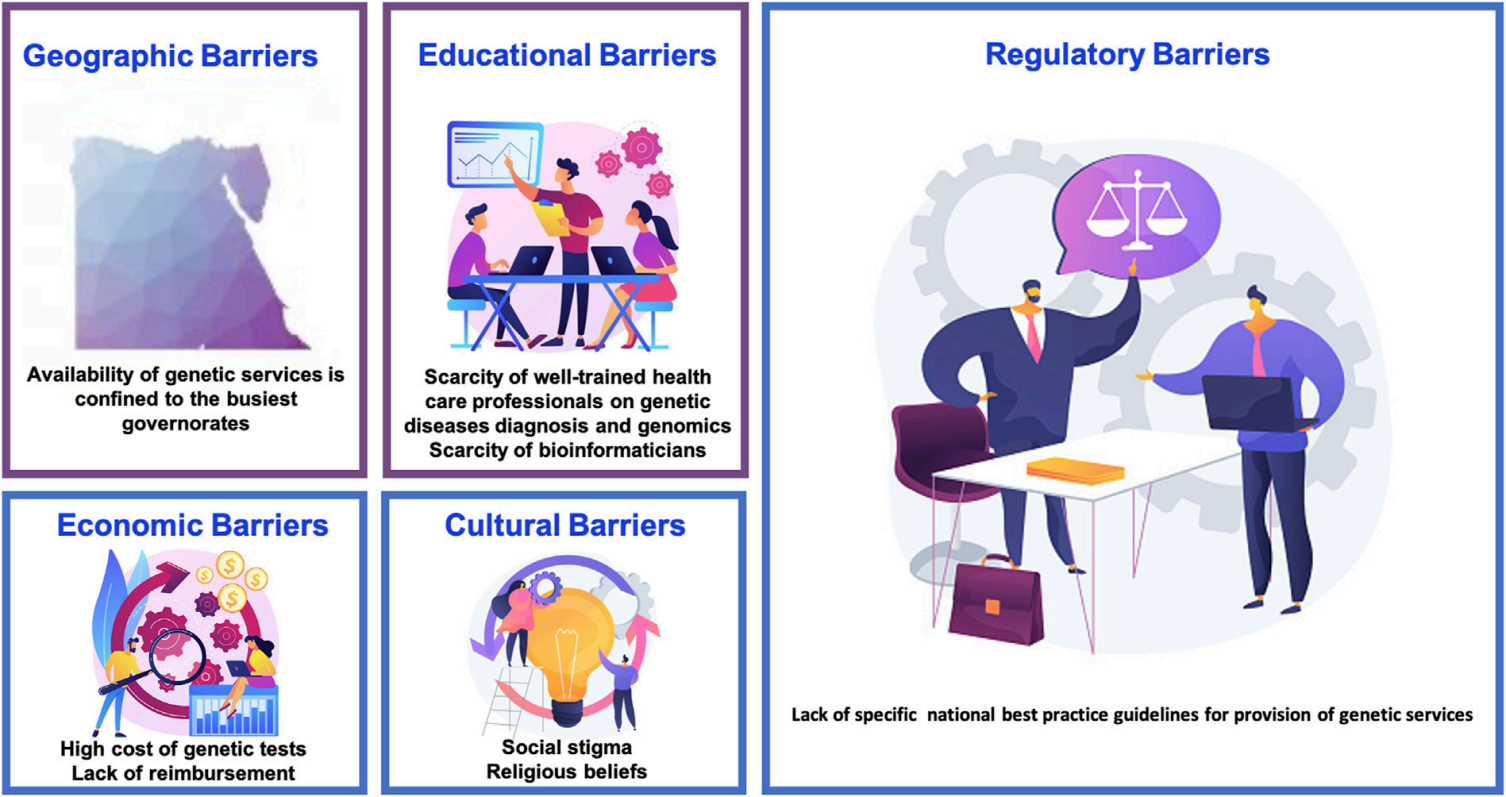
Barriers to genomic/genetic services in Egypt.

The huge leap in genomic information and applications is continuing to push the research efforts and regulations worldwide to keep pace with new inputs and information processing. Genomic medicine impacts almost every aspect of medical practice starting from preconception carrier risk through prenatal genetic diseases diagnosis and newborn screening to cancer risk prediction, precision medicine and gene therapy. Such an ever-growing field mandates continuous training, close monitoring and regulating as well as re-examining data gathering ethics and results conduction methods to patients and families. Identification of challenges is fundamental to improving genomic services as well as establishment of strategic plans to fill the gap towards advancement (Figure 3).

#### Service Availability and Resources

Availability of genetic and genomic services remains a big challenge in Egypt. Although reasonably available in urban areas, rural areas might not have easy access to those services. Moreover, the financial aspect is a fundamental obstacle especially for rural areas, given its high cost and insurance coverage issues. Private sector remains a key player in providing such services, however, some tests are not yet readily available and are outsourced outside the country, which makes the service even more expensive, nevertheless the low demand on such services makes the situation more challenging.

#### Geographical Barriers and Access to Genetic Services

Most University and institutional genetic services are free of charge or of low cost. The national health insurance organization which covers elementary school students provides reimbursement for investigations as well as treatment and rehabilitation services. However, the coverage of genetic tests by the public sector is limited and services for low-income citizens are frequently provided through donations from NGOs or charity. Genetic services provided by the private sector are covered mainly by out-of-pocket payments or reimbursed by private health insurance companies.

A huge step towards standardization, improvement and coverage of healthcare services is expected after the establishment of the new health insurance system in Egypt. Founded in 2019, it will be implemented over six phases to include all governorates and every Egyptian. It aims at maintaining health records for all patients with coverage of most of the service costs and availability of services even in private sectors. Healthcare service providers shall be monitored and accredited by the corresponding accrediting body to ensure quality of service (Gov.eg, 2022).

#### Economic Barriers

The validation of laboratory genetic testing requires the lab to join an external quality assurance (EQA) program, which is usually an international one and difference in currency makes the registration in these programs of high cost. Moreover, the expensive genetic analyzers and kits all add to the cost of genetic testing, rendering it unaffordable for a large sector of the population. Lack of reimbursement is also an issue that faces a large portion of patients and the dependence on out-of-pocket services.

#### Population Acceptance and Cultural Barriers

An important aspect of conducting genetic testing is public acceptance of decisions relying on those test results, otherwise the whole benefit from testing would be questionable. A study conducted by Elgawhary et al. (2008) offered 30 Egyptian females with high-risk pregnancies for beta thalassemia DNA sequencing for fetal tissue prenatally. Out of 22 cases with affected fetuses 14 couples refused to terminate pregnancy reflecting cultural and religious beliefs (Elgawhary et al., 2008) Fear of being socially stigmatized for using genetic services or having a genetic condition as well is particularly common in rural areas, highlighting the cultural considerations for Egyptians (Raouf, 2008).

Public awareness of genetic testing importance and applications should be addressed in media as well as incorporating religious entities to refute any erroneous cultural or religious beliefs that might hinder healthcare conduct.

#### Educational Barriers

Genomic medicine is a relatively new field, knowledge of its importance and application is a cornerstone in providing health care. However, there is still a gap between research studies, application and attitude towards this field in Egypt. Despite the mounting research efforts as aforementioned in the article, physician awareness and knowledge of genomic applications still needs improvement. A cross sectional study by Nagy et al. (2020) was conducted in one of the largest children’s cancer management hospitals; Children’s cancer hospital 57357 through a survey questioning knowledge and attitude towards pharmacogenetic testing. It highlighted a lack of knowledge of pharmacogenomics and test unavailability due to limited funding. The study attributed defective knowledge due to non-integration of such fields in the curricula of undergraduates and postgraduates as well as lack of training and funding (Nagy et al., 2020).

#### Communication and Regulation

One of the pillars of genomic medicine and healthcare management is reporting and communicating test results. However, it is particularly complex in genomic testing settings. It could be partially attributed to the fact that some results could be ambiguous, providing uncertainty more than answers such as reporting of variants of uncertain significance. Such result reporting and communicating to patients is indeed challenging and a well -trained counselor should conduct such results within the frame of a guideline on how to deal with such results (Medendorp et al., 2020) A national standard should be present to set guidance for who to conduct results and how.

“The right not to know'' remains a great ethical concern and an area of conflict when it comes to reporting and communicating secondary or incidental findings that would affect management of patients. Also, communicating results that would affect family members as well as the patient. Patients could choose not to know such important data, leaving the physician in a difficult situation that needs ethical or legal consultation (Marchant et al., 2020). Such situations need to be identified, addressed, and regulated with thorough training of physicians and the judiciary system by the Egyptian government. Nevertheless, regulating direct to consumer genetic tests availability, reporting and result communication and reliability is of equal importance as well.

Complex testing procedures and reports should be governed by an accrediting body and continuously monitored starting from test selection by setting criteria for diagnostic versus research-based tests, what should be offered and what should not be offered, result reporting and communication. General authority for healthcare accreditation and regulation (GAHAR) was established in 2018 under law No. Two for that year, it provides quality standards for laboratories and healthcare facilities by setting standards and inspection services. Currently 19 healthcare facilities are accredited, and many are awaiting accreditation. Such a great step towards standardization and improvement of quality in healthcare in Egypt will reshape health service and give hope to tackling such important aspects of genomic testing in the country (The General Authority for Health Accreditation and Control, 2022).

Establishing national policy initiatives on genetic services is essential. Some laboratories follow the internationally published guidelines as those of the American College of Medical Genetics and Genomics (ACMG), however it is essential to point out that the needs and priorities in developing countries are to a great extent different than those in developed countries (Mohamed, 2015). An important progress by policymakers was in 2020 when the Egyptian parliament approved two important laws that can benefit genetics research as well as genetics services: the data protection law (Privacylaws.com, 2022) and the clinical medical research law (Egypt Independent, 2020). However, the lack of specific national best practice guidelines for provision of genetic services constitutes a major problem in providing high quality genetic/genomic testing services as well as high impact academic research.

### Current Status and Improvement Efforts

The Egyptian MoH&P was keen on implementing a holistic plan related to the upgrading of genetic services in Egypt with special consideration to the prevention and early management of disabilities via addressing three important dimensions:

- Creating registries through an integrated system for treatment and rehabilitation.
- Various programs for prevention and early detection of diseases as (World Health Organization, 2021) premarital care counseling; (Shawky et al., 2011) motherhood program covering all stages from antenatal care, childbirth care, neonatal care and post-natal care (with a focus on congenital anomalies, and early detection of causes of mental retardation); (Hawass et al., 2010) newborn screening program; (Kozma, 2008) childcare program for monitoring child growth and development, immunization and nutrition.
- Supporting rehabilitation centers and introducing the concept of community-based rehabilitation (CBR).

Egypt has also increased investments to fund research in medical genetics and genomics. The main funder is the governmental sector from the ministry of higher education and scientific research *via* the Science, Technology and Innovation Funding Agency (STIFA) and Academy of Scientific Research and Technology (ASRT).

The African Society of Human Genetics (AfSHG), founded in 2003, aims to address the public health burden of diseases across the continent and to develop a road map for translating genomic knowledge across the continent. Through this society, a Pan-African research consortium was established, namely The Human Heredity and Health in African Consortium (H3Africa). It was funded largely by the NIH in the United States and the Wellcome Trust in the United Kingdom and aimed to focus on human health–related genomics and genetics research in Africa. H3Africa funded the implementation of large-scale genomic technologies through collaborative centers in 27 African countries (http://h3africa.org) (El-Kamah et al., 2020).

The “African Genomic Medicine Training Initiative, established in 2016, provides Africa-wide virtual distance learning genomic medicine courses with the aim of developing competencies in Genomic medicine and exploring its practical application targeting African health professionals (H3abionet.org, 2022). The course is hosted by different African countries including pre-recorded lectures & supported by local facilitators linking attendees to the trainer Since 2017, increasing number of ‘‘classrooms’’ are joining this initiative. In 2022, the third iteration was hosted by 27 classrooms in 11 countries, five of these classrooms were in Egypt (Kasralainy hospitals, Cairo University, Suez Canal University, Faculty of Medicine, Modern University for Technology and Information, Faculty of Medicine, Galala University, Faculty of Nursing, Ain Shams University). Egyptian classrooms were the second most frequent to host the course after Nigerian classrooms (Nembaware and Mulder, 2019). This shows the increased interest among Egyptian health professionals to learn about Genomic medicine and to build a capacity of Egyptian trainers who can develop further genomic courses tailored for the Egyptian context in terms of disease burden and available genomic health care services.

The tenth conference of the AfSHG was held in partnership with the National Society of Human Genetics in Egypt and the H3Africa Consortium in Cairo in November 2017. An important outcome of these young research forums (YRFs) at the AfSHG meetings is the development of formal and informal networks to enhance relationships and develop future partnerships among emerging researchers. In the same context, through the 3rd Conference of the Chemical Pathology department, Medical Research Institute, Alexandria University in 2019 a scientific collaboration channel was created with Stellenbosch University in South Africa supported by the Technology Innovation Agency in South Africa through a travel grant. The common interest was implementation of personalized medicine in Breast cancer. Research performed over more than a decade in South Africa (136) led to the development of a rapid BRCA1/2 founder/recurrent mutation assay for improved clinical management of breast cancer and associated co-morbidities. Their vision seeks to move basic research to translation by up-scaling of clinical uptake and implementation of point-of-care genetic testing using an adaptive pathology-supported genetic testing (PSGT) framework (Kotze et al., 2013; Kotze, 2016; van der Merwe and Kotze, 2018). Much of the work in health-related genomics on the African continent is currently being carried out in the context of research. However, to ensure health benefits, advances in diagnosis and treatment of cancer and other NCDs need to shift towards implementation in healthcare settings. In November 2019, the Technology Innovation Agencies in South African and Egypt signed a collaboration agreement that is intended to further enable the promotion of targeted market-oriented research cooperation and technology innovation partnerships between the two countries.

The PSGT framework for moving to a translational phase was implemented as a case study in South Africa. This framework is based on providing a personalized patient experience which aims to distinguish between inherited and environmental causal factors such as lifestyle (epigenetic) or therapy-induced (pharmacogenetic) NCDs for risk stratification and clinical management of multi-factorial and polygenic disorders (Policy Paper: A Framework for the Implementation of Genomic Medicine for Public Health in Africa, 2022). Genomic medicine requires expensive laboratory equipment, and it may take many days to weeks for result generation. In a resource limited African setting such as Egypt, this could lead to loss-in-follow-up and thereby ineffective risk management. The South African experience from using rapid, cost-effective point of care (PoC) DNA assays to speed up the process of genetic testing and decrease cost demonstrated the feasibility of this approach in a pilot study (Mampunye et al., 2021) POC *BRCA1/2* testing combined with genetic counselling to inform patients about the limitations and benefits of mutation-specific PoC tests versus comprehensive sequencing methods will increase access to genomic medicine, given the clinical dilemma caused by a relatively high frequency of variants of uncertain clinical significance (VUS) uncovered by gene panel testing and whole exome/genome sequencing (WES/WGS). PSGT is used to identify the target group most likely to benefit from germline DNA testing and/or tumour gene profiling and facilitate clinical interpretation of genetic results to add value in combination and beyond pathology test results. Incorporating WES/WGS as the research component of PSGT provides clinicians with the option to request additional genomic information pertinent to the patient (Policy Paper: A Framework for the Implementation of Genomic Medicine for Public Health in Africa, 2022; Kotze, 2016; van der Merwe and Kotze, 2018).

There have been a few studies in Egypt aiming for a similar approach by associating serum 25 (OH) Vitamin D level and breast cancer prognosis (El Shorbagy et al., 2017; Ismail et al., 2018; Abdel-Razeq, 2019). However, we need to shift from efforts done in a research context to actual implementation in a healthcare setting. A pipeline moving from primary research findings to validation of results in a wide population segment to translation into clinical application is required. Application in routine diagnostic, prognostic and therapeutic care should be followed by population level implementation.

### The Reference Genome Project for Egyptians and Ancient Egyptians

**FIGURE 4:**
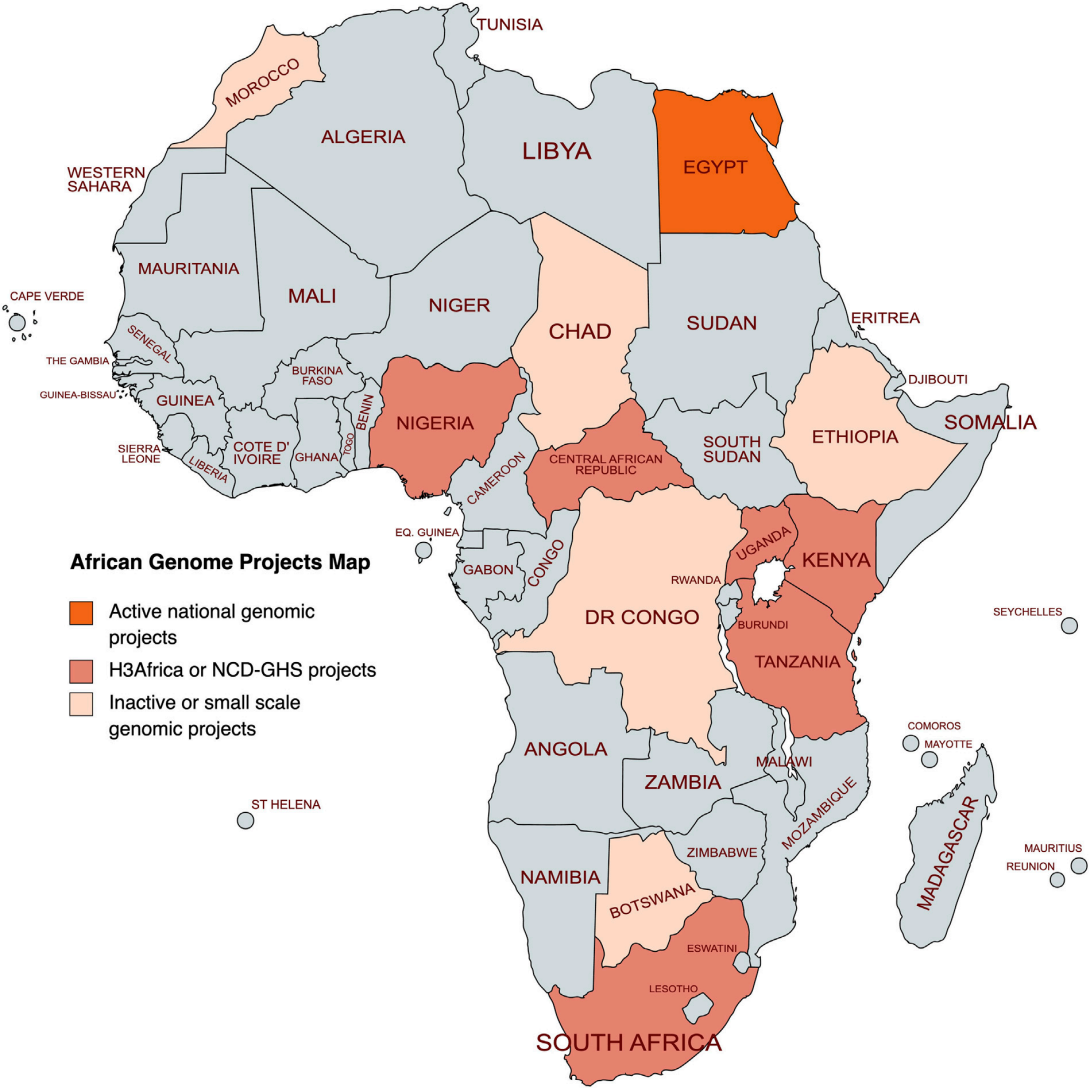
African genome projects map. Created with mapchart.net.

In 1990, the first Human Genome Project worldwide was started aiming to decode the sequence of the human genome (Watson, 1990; Cantor, 1990). After 8 years, the Icelandic deCode Project was initiated to link genomic data with other medical and non-medical data (Palsson and Rabinow, 1999). In 2010, the UK10K project was announced for where it established a collaboration among many United Kingdom public and private entities, to identify genetic causes of rare diseases (The International Genome Sample Resource, 2022). In 2015, the United States and China started their large precision medicine initiatives based on genomic medicine (Cyranoski, 2016; Stark et al., 2019; All of Us Research Program, 2022; Globaltimes.cn., 2022). In 2018, Europe announced an initiative entitled “Towards access to at least 1 million sequenced genomes in the EU by 2022” aiming to share genomic information and best practices among the European union countries (Saunders et al., 2019; 1+ Million Genomes, 2022). All these major genomic initiatives were far away from Africa but owing to the importance of decoding the African human genome, efforts and collaborations have started to achieve this purpose since 2010 via establishing the H3Africa international project. According to our literature search through the Egyptian presidential online resources and the different African genomic initiatives websites, a total of 13 African countries were found to have either genomic projects and/or genomic databases among the 54 countries constituting the African continent (Kovanda et al., 2021), of which only the Reference Genome Project for Egyptians and Ancient Egyptians is currently active (Kovanda et al., 2021; Ain Shams University Official Website, 2022) (Figure 4). The remaining 12 projects were either not currently active or were part of the H3Africa international project (Adoga et al., 2014; Ramsay, 2015) or the Nigerian 100K Non-Communicable Diseases Genetic Heritage Study (NCD-GHS) (Genomeweb.com., 2022) and hence cannot be classified as a national project. In Nigeria, beside the NCD-GHS study, a venture called 54Gene, named to reflect the 54 countries in Africa, has started its activity aiming to build the continent’s largest biobank. The first step in this venture was a study, launched in 2020, to sequence and analyze the genomes of 100,000 Nigerians with a promise of bringing precision medicine to Nigeria (Maxmen, 2020; Genomeweb.com., 2022).

In Egypt, the current National Reference Genome Project for Egyptians and Ancient Egyptians was preceded by a smaller scale project “EgyptRef” in 2018 with the aim to establish a reference genome for Egyptian and North African populations to complement the Genome Reference Consortium human genome (GRCh) (Egyptian-genome.org., 2022). The most important details and comparisons related to the previous and the current Egyptian genome projects are clarified in Table 1 and compared also to the major African genomics endeavors in Africa which is H3Africa International genome Project. Currently, there are high expectations on the National Reference Genome Project for Egyptians and Ancient Egyptians in terms of entering the era of precision medicine to Egypt, improving cost-effective diagnostics, with more targeted prevention and treatment for Egyptians.

**TABLE 1:** Comparison between the current genomic projects in Egypt and Africa.

	Pilot egyptian human genome project (EgyptRef)	The national reference genome project for egyptians and ancient egyptians	H3Africa international Genome project
Duration	2018 and ongoing	2021–2025 (first phase)	2010 and ongoing
Aims and scope	To establish a reference genome for Egyptian and North African populations to complement the Genome Reference Consortium human genome (GRCh)	To establish a benchmark genome center for creating the map of the Egyptian human genome to discover and accurately determine the genetic characteristics of various diseases aiming to help Egypt in entering the era of precision medicine.	H3Africa empowers African researchers to be competitive in genomic sciences, establishes and nurtures effective collaborations among African researchers on the African continent, and generates unique data that could be used to improve both African and global health.
		The project’s scope is classified into three categories:	
		A- The population genome	
		B- The Genome of the ancient Egyptians, and C- Diseased genome.	
Website	https://www.egyptian-genome.org	Under Construction	https://h3africa.org
Coordinator	The genetics and systems biology divisions of LIED, Lübeck University, Germany	Medical Research and Regenerative Medicine Center at the Ministry of Defense, Egypt	The African Society of Human Genetics
Partners	The Center for Experimental Medical Research (MERC), Mansoura University, Egypt	Thirteen Temtamy and Hussen, (2017) Egyptian universities and research centers from the Ministries of Defense, Higher Education, Scientific Research, Health and Communications, and a number of civil society institutions.	The National Institutes of Health (NIH)
			The Wellcome Trust (WT)
			The African Academy of Sciences (The AAS)
Funding	The German Science Foundation, excellence program (EXC 306) and the DAAD	Egyptian governmental funding a total of 62,500,000 dollars for first phase over 5 years	NIH Director’s Common Fund. Till now, H3Africa activities has been supported with 176 million dollars investment by NIH/WT
Data sharing	Public access	No data Yet	Public access

As one of the main goals of this huge national project is to upgrade the infrastructure of genetics and genomics in Egypt, the project acquired a number of NGS platforms and formed a network with previously existing platforms in different universities or research centers which will enable the project to identify enormous number of causal mutations in a short period of time and at relatively low cost. The availability of sequence variants from the pilot EgyptRef project started in 2018 will also be made use of when an available reference genome is required. In this way, NGS based genetic screening will be possible for the identification and mapping of causal mutations among the Egyptian population. However, data obtained from NGS is highly complicated and requires more sophisticated data processing (Pereira et al., 2020). NGS based genetic approaches are vastly reliant on compatible bioinformatics tools and pipelines to obtain the final outputs and desired information. Numerous bioinformatics tools and pipelines have been developed to perform the different functions. Utilization of these tools/pipelines depends on the NGS methodology, type of experimental materials, and the genome of organisms (Sahu et al., 2020). Many Egyptian bioinformaticians are sharing their experience in this national project and will help in all analysis phases.

Advances in sequencing technologies provided the world with a great tool to decode the human genome efficiently, at much lower costs than before and at a significantly faster rate. Having said that, still the reference genome lacks population specific variations. A great milestone in advancing health care services was achieved by the establishment of the “Egyptian reference human genome” through sequencing Egyptian individuals’ genome, a project conducted by Mansoura University in collaboration with Lübeck university and the German science foundation, DFG, excellence program (EXC 306) and DAAD, www.egyptian-genome.org. The results of the sequencing showed Egyptian specific gene variants, paving the way towards precision medicine implementation and adding greater value for genetic research as well (Wohlers et al., 2020). The next step that is underway is the implementation of “Reference Genome Project for Egyptians and Ancient Egyptians”, which should aim at further characterization of the genetic variants in Egyptians and evaluation of their impact and relevance to diseases.

On the 31st of January 2022, a detailed disclosure of the Egyptian genome project was revealed at an event “The Reference Genome Project for the Egyptians and the Ancient Egyptian” held at the Dubai Expo 2020, United Arab Emirates. The event was organized by the Egyptian Ministry of Higher Education and Scientific Research, represented by the Academy of Scientific Research and Technology ASRT, in partnership with the Sheikh Zayed Center for Genetic Research, affiliated with the Emirates Society for Genetic Diseases. It was announced that according to the executive plan, the implementation of the project will end by the end of 2025, with an initial cost of two billion Egyptian pounds (128 million dollars).” With the first phase starting with a sample of 20,000 Egyptians to identify the most common diseases, and then increasing to 100,000 over 5 years for the first phase, until the project covers all of Egypt. The ASRT began implementing the recommendations of the specific councils in the academy to launch the reference genome program for the Egyptians, in cooperation with the Center for Research and Regenerative Medicine Center (ECRRM) of the Armed Forces being the main research body, and a number of scientific and executive bodies in the Egyptian state are participating in the project as well, represented by the Ministries of Defense, Health and Communication, more than 15 universities, research centers and civil society institutions (Teller Report, 2022; Egypt Center for Research and Regenerative Medicine, 2022; Emirati Foundation, 2022; Archyde, 2022; Newsbeezer, 2022).

### SWOT Analysis of Current Genetic/Genomics Services in Egypt

**FIGURE 5:**
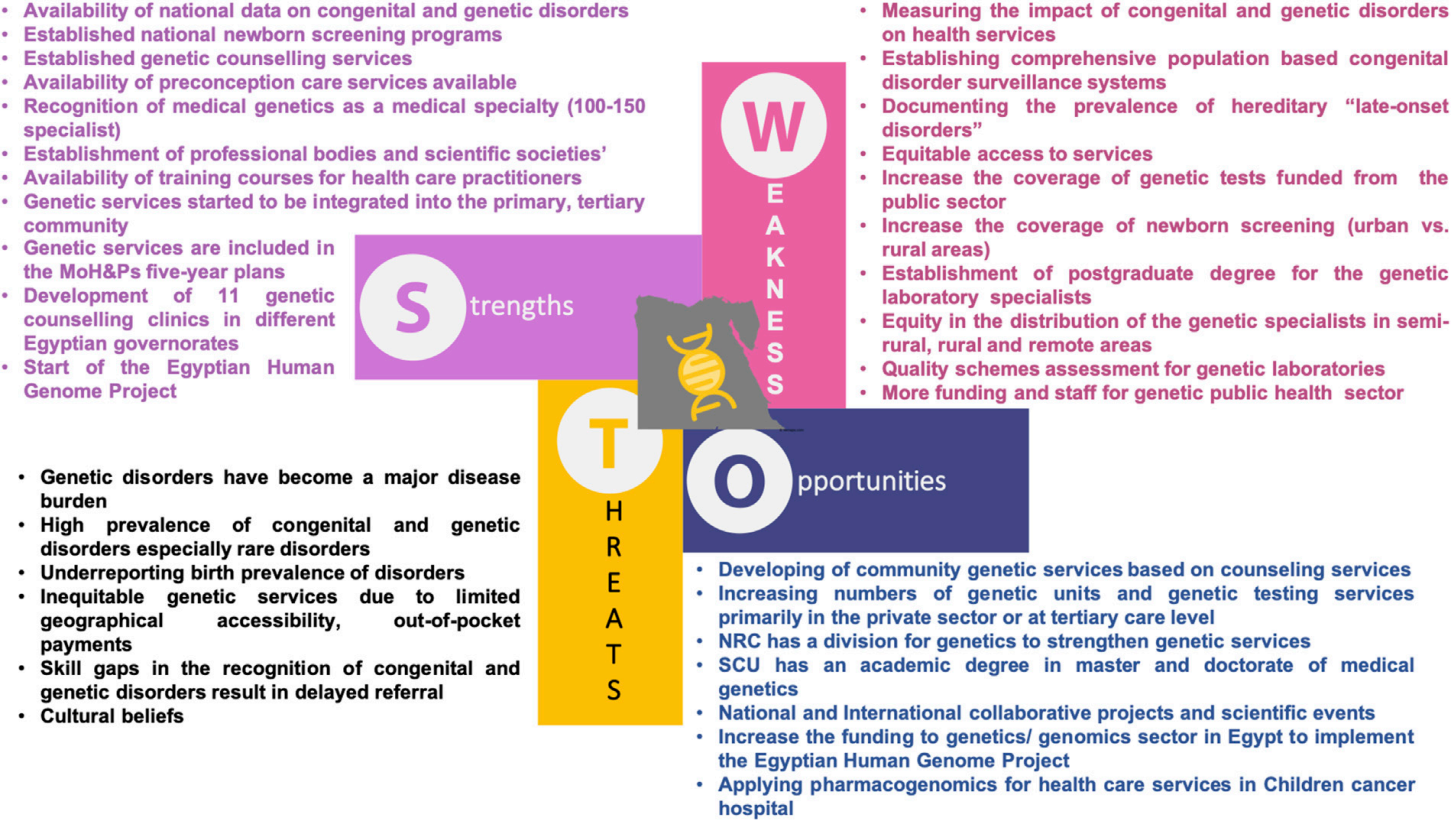
SWOT analysis of current genetic/genomics services in Egypt (MOH&P: Ministry of Health and Population; NRC: National Research Center; SCU: Suez Canal University).

The presented SWOT analysis (Figure 5) is based on the results of the Erasmus project report entitled “Genetic Testing in Emerging Economies (GenTEE)” in the period from 2010 to 2012 (Genetic Testing in Emerging Economies (GenTEE) Project, 2022). The GenTEE partners were from European and non-European partner countries, where Egypt was among the partners in this project. The GenTEE report relied upon implementing a wide range survey in each partner country based on a common method/framework for data ascertainment, thus allowing examination and comparison of the following: Service development in relation to the existing health care systems, available genetic services resources, national genetic testing services within the available health policies, and factors that affect the state of genetic service delivery such as the demographic, socio-economic and legal factors. This survey addressed nine dimensions using a set of indicators pre-defined by the project’s consortium which were: demography and health indicators, health expenditure and financing, indicators of congenital/genetic “disorder burden”, availability of genetic services, access to genetic services, state of genetic services, research priorities in genetics/genomics, patient organizations and public education in genetics, and future outlook for service development in each country. The survey targeted specific outcomes specifically genetic testing services within the health care settings, which were: PND and PGD, newborn screening, carrier screening, diagnostic testing for congenital and genetic disorders, including testing for common disorders with a major single gene subgroup, pharmacogenetic testing, and genetic susceptibility testing (e.g., for infectious diseases). For data ascertainment purposes, the data collection of the survey was based on published data including grey-literature, accessible unpublished reports/data, and expert opinion. Before a country report was accepted by the GenTEE consortium it had to be submitted to an external expert review for validation.

From this GenTEE report, it was obvious that the urban, upper-middle classes in most developing countries including Egypt are the main beneficiaries of the genetic services. This is mostly attributed to the economic class of this category, where they can afford these services in the private sector. Also, the scarcity of health care professionals specialized in genetics is another important problem facing the genetics services in Egypt.

In addition, we have a huge mismatch in Egypt between the non-availability of enough services in the genetic/genomic field and in developing research capacities for improving the Egyptian patients care. Therefore, to achieve a rapid development of genetic/genomic Egyptian services, the current service infrastructure needs to be upgraded in Egypt. This step will support the successful driving of genetic/genomic facilities and academic research into accredited reliable services and will thereby be reflected on the overall population health outcomes.

### Multidisciplinary Collaboration for Advancing Genomics in Egypt: Recommendations and Future Directions

**FIGURE 6:**
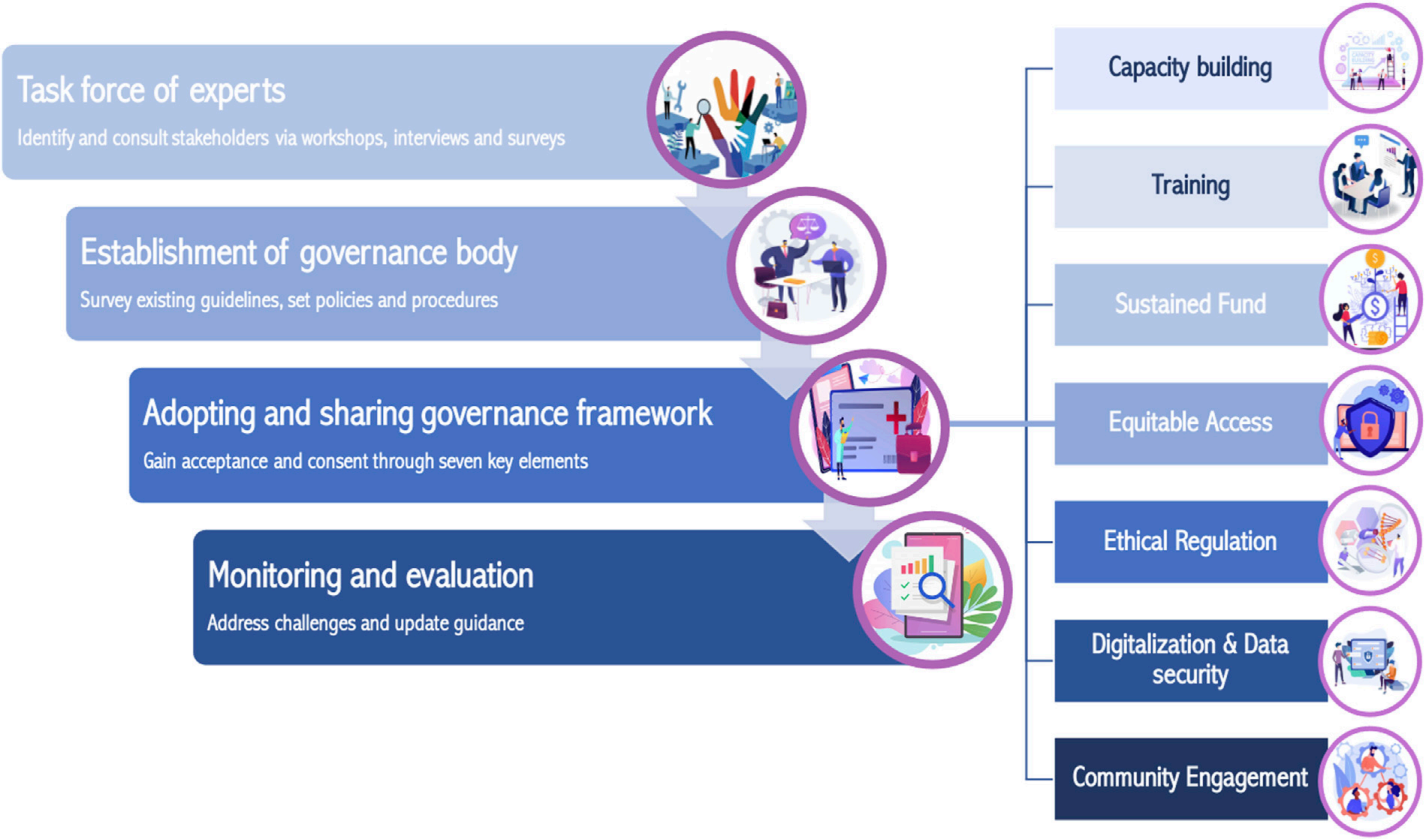
Suggested stepwise approach for genomic medicine governance establishment.

A multi-disciplinary approach in the field of genomics is currently emerging world-wide. This approach for ensuring engagement, informed decision-making and supporting clinicians has been made possible by rapid advances in DNA sequencing, bioinformatics and digital applications. All these evolving technologies along with cornerstone directions and initiatives represented in the “Egyptian genome” and the “comprehensive Egyptian health insurance” projects recently adopted presidentially in Egypt paves the way for successful genomic medicine integration in healthcare system in Egypt. Multidisciplinary collaboration is the mainstay of genomic medicine integration and we recommend it to be implemented under the umbrella of governance body (Adebamowo et al., 2018) in a stepwise approach (Figure 6). A task force of experts in the field of genomics is recommended to be assigned to identify and consult stakeholders (Tindana et al., 2019). This pivotal task is a principal key for acceptance, consent and collaboration, ensuring successful implementation. Consultation with stakeholders could take place in different forms such as workshops, interviews and/or surveys to form a multidisciplinary governance body (Tindana et al., 2019). Inclusion of all stakeholders in the governance body is advisable for lean, resistance free implementation. Stakeholders could include the Ministry of Health and Population which is a main primary care provider, Ministry of Higher education and Research, a main training and research entity, Egypt Center for Research and regenerative Medicine (ECRRM), a main contributor in the Reference Genome Project for Egyptians and Ancient Egyptians, as well as investors, religious and judiciary entities, non-profit organizations and civil societies.

The governing body should set policies and guidelines for every sector involved in genomic medicine establishment and integration in healthcare as well as adopting genomic surveillance strategy for pathogens with pandemic or epidemic potentials. Sharing guidelines with international and national communities (Tindana et al., 2019) for broader acceptance and refinement is advisable and could be established through conferences and meetings. The governing body should address the needs of the Egyptian community, respecting the cultural and religious beliefs, avoiding stigmas and harm, addressing fears and establishing genuine Egyptian genomic medicine services. It should practice as well good governance principles; accountability, transparency, responsiveness, equitability, inclusiveness, consensus oriented, effectiveness, efficiency, follow the rule of law and participatory (Governance for Sustainable Human Development, 1997). We recommend that the governance should adopt a framework (Policy Paper: A Framework for the Implementation of Genomic Medicine for Public Health in Africa, 2022; O’Doherty et al., 2021) that fulfills the above-mentioned principles as well as be inclusive of key elements for genomic medicine establishment. We suggest the following elements to be considered in the framework.

### Key Elements to be Considered in the Governance Framework

**Capacity building** (Yakubu et al., 2018) is the main pillar of genomic medicine establishment and should be planned for, governed and monitored. The governance should ensure the presence of laboratories with latest technologies as well as fully functional **biorepository** that will house all the biological specimens that will be collected. This infrastructure will ensure that biological specimens remain in the country and will encourage large-scale collaboration between Egyptian scientists while fostering collaborations between Egyptian and international scientists as well.

Infrastructure and technology are particularly important in a high paced, rapidly evolving field such as genomics, yet alone never ensure needs are met to population. Complex technology and huge amount of data generated mandates high level of expertise in several fields such as; genetic counseling, bioinformatics and technical operation of various instruments. Investment in individuals through **training** (Policy Paper: A Framework for the Implementation of Genomic Medicine for Public Health in Africa, 2022) is the mainstay for sustainable and high standard quality of service. It could be achieved through scholarships for post graduate students, training programs and workshops for physicians, data scientists, scientists and genetic counselors in collaboration with experts in the field from local and international universities. Training program should be in multiple disciplines, including genomics (high-throughput technologies), genetics, epidemiology, bioinformatics, statistical genetics, and Ethical Legal and Social Issues (ELSI).

Integration of introductory genomic medicine course in undergraduate medical student curriculum as well as establishment of genomic medicine and bioinformatics post graduate master’s degrees is important to prepare good calibers with the needed expertise to deliver the best of service. However, encouraging young physicians and scientists to hold relevant degrees could be challenging, especially if there is not a rewarding salary, opportunities for hiring in the system are scarce as well as few mentorships available as in the case of African countries (Policy Paper: A Framework for the Implementation of Genomic Medicine for Public Health in Africa, 2022), such concerns should be identified and addressed.

The governance should **encourage research** in genomics and **raise funds** allocating them towards this field and promote collaboration among different entities and investors under its auspices, as well as setting financial plans for sustainable funding (Cazabon et al., 2021) of genomic medicine related services and research. Moreover, it should facilitate and encourage sharing expertise with universities and organizations around the globe.

The governance should support and facilitate collaborative efforts to integrate genomic medicine service in the healthcare system including the public and private sectors, monitor performance and identify challenges. Ensure thorough training to all involved personnel as well as public genomic medicine literacy for efficient and effective deployment of service.

Although genomic medicine is present in the medical field for years now, the service remains highly expensive especially for low-income societies, to ensure **equitable access**, insurance system should cover expenses. Reimbursement of service should be for both public and private sector with cost predefined and controlled by the governance. Also, the type of service covered by insurance, whether testing, treatment or counseling according to preset tiers defined by the governance, are equally applied on both public and private sectors. Having insurance coverage to private sectors as well shall expand the availability and accessibility of genomic medicine, decreasing the burden on public sector and better allocating ministries’ budget to other demanding sources. The new comprehensive insurance system to be established in Egypt is a fundamental step towards advancing genomic medicine integration in healthcare system.

In the digitalized era and with the ever-growing populations, paperwork became more and more difficult to catch advancements in several fields. For easier, rapid and standardized archiving, simple and fast access to data and reaching out to people in underprivileged areas, **digitalization of service** is indispensable.

Reaching out for patients remotely is widely spread nowadays through health applications that offer simple information, appointment booking, test results and immunity passports as in Covid-19 pandemic nowadays in certain countries. Applications are emerging to help patients receive their genomic test results with greater understanding of the nature of test, result, actions to be taken, impact on health and family, giving them choice of what information to receive or not. Genomics ADvISER (Bombard et al., 2018) and Genetics Navigator are examples for digital applications offering guidance in incidental findings and entire genomic testing respectively (Bombard and Hayeems, 2020). Although fast, readily accessible, updated and can notify patient with updates, alone it is not enough to deliver all information, consult and conduct result. In-person meeting with genetic counselor would be essential in Egypt to ensure good understanding and provide emotional support.

Digitalization of service either through hospital, clinic information systems, medical records, applications and websites, generate enormous amount of **data that should be secure and confidential** (Policy Paper: A Framework for the Implementation of Genomic Medicine for Public Health in Africa, 2022) under law and its abidance must be monitored. Legislation and enforcement of this law is the responsibility of the governance in collaboration with the judiciary and legal entities.

For community acceptance, service must be assured it is within the **religious and ethical frame** (Policy Paper: A Framework for the Implementation of Genomic Medicine for Public Health in Africa, 2022) of the community. Formulation of tiers of contests (broad, tiered, conditioned, restricted) to the usage and sharing of data and specimens for research purposes gives more confidence and acceptance in genomic medicine services and research especially if the archiving is within the country. Governance should collaborate with religious entities as well to encourage community to endorse genomic medicine and assure adherence to values.

Encouraging and supporting the establishment of civil societies promoting **community engagement** (Cazabon et al., 2021) in decision making, addressing their needs and resolving challenges. Such efforts would promote community acceptance bringing consensus and ease implementation.

**Monitoring and evaluation** is indispensable for a project to be successful and sustainable, the governance should continuously monitor and evaluate the establishment of genomic medicine to identify challenges and update guidance accordingly.

## Conclusion

Genomic medicine is a new emerging field in clinical care, having large impact on communicable diseases, as seen in the ongoing pandemic, as well as pharmacological treatments, rare and undiagnosed diseases and oncology. There is enormous amount of genomic data that is generated continuously from around the world. However, data from other countries could lack special features that are specific and relatable to Egypt and African countries. The recent launch of Reference Genome project for Egyptians and Ancient Egyptians represents a new era of medicine in Egypt and is a corner stone in genomic medicine establishment. In this review we presented the needs of our community as regards to genomic medicine in several fields, pointing out the challenges and barriers towards advancing this type of clinical care into our healthcare system. We also suggested establishment of a governance body which would be essential in regulating the genomic medicine services in Egypt. The suggested governance body would improve the existing regulatory process, develop and periodically review guidelines and policies as well as collaborating efforts among stakeholders. Observing the thirst for genomic medicine in Egypt, it is hopeful that a revolutionary era of healthcare service would be seen in the country soon.
